# Collaborative Action on Fetal Alcohol Spectrum Disorder Prevention: Principles for Enacting the Truth and Reconciliation Commission Call to Action #33

**DOI:** 10.3390/ijerph16091589

**Published:** 2019-05-07

**Authors:** Lindsay Wolfson, Nancy Poole, Melody Morton Ninomiya, Deborah Rutman, Sherry Letendre, Toni Winterhoff, Catherine Finney, Elizabeth Carlson, Michelle Prouty, Audrey McFarlane, Lia Ruttan, Lisa Murphy, Carmen Stewart, Lisa Lawley, Tammy Rowan

**Affiliations:** 1 Centre of Excellence for Women’s Health, Vancouver, BC V6H 3N1 Canada; NPoole@cw.bc.ca; 2 Canada Fetal Alcohol Spectrum Disorder Research Network, Vancouver, BC V5R 0A4, Canada; 3 Centre for Addiction and Mental Health, London, ON N6G 4X8, Canada; melody.mortonninomiya@camh.ca; 4 Nota Bene Consulting Group, Victoria, BC V8R 1P8, Canada; drutman@uvic.ca; 5 School of Social Work, University of Victoria, Victoria, BC V8P 5C2, Canada; 6 Alexis Nakota Sioux Nation, Glenevis, AB T0E 0X0, Canada; fasd@ansn.ca; 7 Stó:lō Service Agency, Chilliwack, BC V2R 4G5, Canada; Toni.Winterhoff@stolonation.bc.ca (T.W.); Catherine.Finney@stolonation.bc.ca (C.F.); 8 Department of Educational Psychology, University of Alberta, Edmonton, AB T6G 2R3, Canada; emcarlso@ualberta.ca; 9 Skidegate Health Centre, Skidegate, BC V0T 1S1, Canada; michelle.prouty@skidegatehc.ca; 10 Lakeland Centre for Fetal Alcohol Spectrum Disorder, Cold Lake, AB T9M 1P1, Canada; AMcFarlane@lcfasd.com (A.M.); LMurphy@lcfasd.com (L.M.); 11 Independent Scholar, Edmonton, AB T6J 0V5, Canada; lmruttan@shaw.ca; 12 Kermode Friendship Society, Terrace, BC V8G 2N7, Canada; cstewart@kermode-fs.ca (C.S.); llawley@kermode-fs.ca (L.L.); 13 Mount Carmel Clinic, Winnipeg, MB R2W 5L4, Canada; trowan@mountcarmel.ca

**Keywords:** FASD, Two-Eyed Seeing, indigenous knowledge, prevention, alcohol, maternal health

## Abstract

The association between fetal alcohol spectrum disorder (FASD), residential schools and subsequent assimilatory policies in Canada is of such significance that it was included in the groundbreaking Truth and Reconciliation Commission of Canada’s Final Report through Call to Action #33, which focuses on collaboratively developing FASD prevention programs in Indigenous communities. A consensus statement with eight tenets for enacting Call to Action #33 was co-developed in May 2017 using a Two-Eyed Seeing approach during and after a meeting on Indigenous approaches to FASD prevention held in Canada. The consensus statement provides guidance for creating community-based, culture-led FASD prevention programs in Indigenous communities. The eight tenets reflect the diverse perspectives of Indigenous and non-Indigenous participants, are grounded in available research evidence, and align with Indigenous worldviews and wellness models. This paper uses the consensus statement and eight exemplary FASD prevention programs from Indigenous communities and organizations across Canada to highlight identity, culture, and relationships as central elements of FASD prevention in Indigenous communities. The consensus statement provides guidance for developing community- and culture-led FASD prevention programs and highlights the importance of Indigenous knowledge systems in developing and researching FASD prevention in, and with, Indigenous communities.

## 1. Introduction

The physical, spiritual, and emotional wellbeing of mothers, and the meaningful roles of pregnancy and mothering are essential components to preserving Indigenous cultural traditions and languages [1,2]. However, colonial practices including the Indian Act, government and church-run residential schools, historical and contemporary child welfare practices, and systemic violence against women, have disrupted families, communities, and traditional approaches to Indigenous peoples’ health [3,4,5,6]. In Canada, the term ‘Indigenous peoples’ refers to descendants of the peoples of North America who were present before colonization and encompasses three distinct groups: First Nations, Métis, and Inuit. These three populations are comprised of distinct and diverse groups, with unique histories, languages, cultural practices, and spiritual beliefs.

Indigenous peoples and communities were impacted by colonization in many different ways, including through the criminalization of midwifery whereby traditional models for maternal health became illegal and child birth became medicalized. In many communities, women were removed from their community to distant hospitals to give birth. These policies and practices prevented the transmission of birthing and child-rearing practices and perpetuated the tragic and unfounded misconception that Indigenous families and communities were unable to raise their children.

In 2008, the Truth and Reconciliation Commission of Canada (TRC) was established after decades of inadequate responses to the legacies of the residential school system. The residential school system was designed to forcibly assimilate and eradicate Indigenous peoples through the removal of children from their families, communities, languages, and cultures and placing them in government and church-run schools [7,8,9]. Residential schools impacted all aspects of Indigenous peoples’ lives through the disruption of families and communities, loss of land, culture, language, and identity, and perpetuated feelings of shame and loss. The effects were not exclusively experienced by those forced to attend but have been felt across generations. This intergenerational transmission of trauma underlies the risk for alcohol use in pregnancy and has manifested within communities in many ways, including through the desire to self-medicate and high rates of substance use and addiction [7,10,11].

As a result, fetal alcohol spectrum disorder (FASD) has emerged as a health priority within Indigenous communities in Canada. Data on FASD prevalence in Canada is often methodologically diverse and pan-Indigenous, discounting geographic, demographic, linguistic, and cultural diversity of First Nations, Métis, and Inuit and creating challenges for identifying Indigenous-specific FASD prevalence rates [12,13,14]. Although several early prevalence studies showed high rates of FASD among Indigenous peoples, these studies are largely outdated and had significant methodological concerns. The published research has been mainly conducted by non-Indigenous researchers using Western methodologies and epistemologies, which do not integrate Indigenous worldviews or practices and invariably reproduce colonial practices. Several prevalence studies have also been generalized to represent all Indigenous peoples in Canada despite being garnered by a particular nation and/or reserve out of concern for the prevalence of FASD their specific community [12,13,14].

Despite these limitations, the National Collaborating Centre for Aboriginal Health views FASD as a “clear public health concern” among Indigenous peoples [13], which was also reflected in the inclusion of FASD in the TRC’s Final Report (2015) and with Call to Action #33, which states: “We call upon the federal, provincial, and territorial governments to recognize as a high priority the need to address and prevent Fetal Alcohol Spectrum Disorder (FASD), and to develop, in collaboration with Aboriginal people, FASD preventive programs that can be delivered in a culturally appropriate manner” [9] (p. 221).

Call to Action #33 has been a driver of new and renewed action on FASD prevention in Indigenous communities in Canada. The Consensus Statement: Eight Tenets for Enacting the Truth and Reconciliation Commission’s Call to Action #33 (hereafter “Consensus Statement”) is a framework for FASD prevention in Indigenous communities that was developed collaboratively by and with Indigenous and non-Indigenous peoples from diverse communities, institutions, and organizations across Canada. In this paper, we highlight exemplary and current community-based and community-driven FASD prevention efforts in select communities that embody tenets from the Consensus Statement. Each of the programs included in this paper recognizes that reducing substance use in pregnancy and preventing FASD is not just a women’s or mental health issue, but a complex health and social justice concern requiring trauma-, culture-, and gender-informed responses at the individual, family, and community levels. All examples in this paper affirm and highlight the importance of *Etuaptmumk*/Two-Eyed Seeing and the incorporation of Indigenous knowledge systems in developing comprehensive and effective FASD prevention programs.

## 2. Materials and Methods

*Etuaptmumk*, or Two-Eyed Seeing, refers to the alignment of Indigenous and Western knowledges and worldviews as distinct epistemological systems that are woven “back and forth” between the two worldviews without assimilation or supremacy [15,16,17]. *Etuaptmumk* respects and integrates the strengths of Indigenous knowledge and Western science relating to health and wellness and helps to preserve and recognize traditional knowledge while concurrently utilizing the diverse healing and medical practices that are available today [18]. We have made every effort to use *Etuaptmumk* in the meetings described in this paper that led to the development of the Consensus Statement, and in our continued work in community-based and community-driven FASD prevention.

In May 2017, the Centre of Excellence for Women’s Health, Thunderbird Partnership Foundation, and Canada FASD Research Network hosted the Dialogue to Action on the Prevention of Fetal Alcohol Spectrum Disorder (hereafter referred to as “Dialogue”), a one-day meeting on the Unceded Territories of the Coast Salish Peoples. The Dialogue brought together 23 national experts on Indigenous wellness and FASD prevention, including community leaders, frontline workers, policymakers, and researchers, to discuss principles necessary for enacting Call to Action #33 using *Etuaptmumk*.

From the Dialogue, principles for enacting Call to Action #33 were collaboratively developed through a thematic analysis of detailed notes using NVivo, a qualitative data analysis software, which led to the identification of eight key themes. These themes were refined into the Consensus Statement: Eight Tenets for Enacting the Truth and Reconciliation Commission’s Call to Action [19] by interested Dialogue participants. The Consensus Statement was released on June 2, 2017 on the second anniversary of the release of the TRC’s Calls to Action.

In March 2019, a second event, Advancing Collaborative Action on FASD Prevention, was hosted on Musqueam Territory by the Centre of Excellence for Women’s Health in collaboration with the First Nations Health Authority. This workshop brought together 27 program providers, frontline workers, and researchers representing diverse nations and organizations across Canada who are involved in community-based, community-driven FASD prevention programs to explore how participants can inform and support one another in program development, implementation, and evaluation. Several of the programs discussed during the workshop are highlighted in this article and were captured through detailed notes and graphic recordings on how participants are engaging in community-based, community-driven FASD prevention program development, implementation, and evaluation.

This article uses the Consensus Statement, detailed notes from both meetings, and phone interviews and email communication conducted in March 2019, to illustrate how community-based, community-driven FASD prevention programs exemplify and highlight the importance of eight tenets of the Consensus Statement for Indigenous-led FASD prevention.

## 3. Results

### 3.1. Consensus Statement: Eight Tenets for Enacting the Truth and Reconciliation Commission’s Call to Action #33

**Figure 1:**
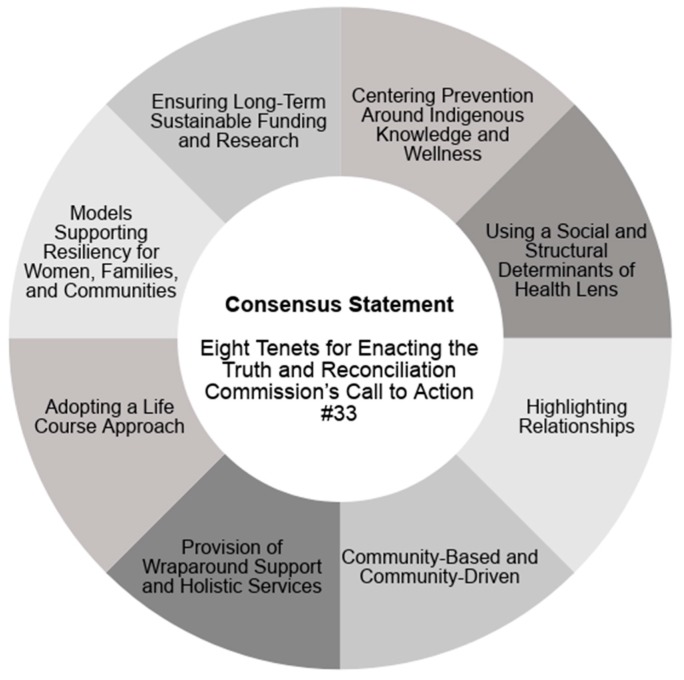
A graphic visualization of the Consensus Statement: Eight Tenets for Enacting the Truth and Reconciliation Commission’s Call to Action #33. The eight tenets are (1) Centering Prevention around Indigenous Knowledge and Wellness; (2) Using a Social and Structural Determinants of Health Lens; (3) Highlighting Relationships; (4) Community-Based and Community-Driven; (5) Provision of Wraparound Support and Holistic Services; (6) Adopting a Life Course Approach; (7) Models Supporting Resiliency for Women, Families, and Communities; and, (8) Ensuring Long-Term Sustainable Funding and Research.

The Consensus Statement aligns with successful approaches to FASD prevention and wellness and can be used to guide the creation of culturally-informed FASD prevention programs, supports, and services for women, families, and communities. The eight tenets of the Consensus Statement are interconnected and directly respond to Call to Action #33 through providing a foundation necessary for interdisciplinary and reconciliatory research action and program planning [19]. The eight tenets are captured in Figure 1.

#### 3.1.1. Tenet #1: Centering Prevention around Indigenous Knowledge and Wellness

FASD prevention efforts should be guided by the concept of wellness and principles of land, lineage, and language; as guided by the First Nations Mental Wellness Continuum Framework and [20] and Honouring Our Strengths: A Renewed Framework to Address Substance Use Issues among First Nations People in Canada [21]. Using the Wellness Framework, prevention efforts can support individuals, families, and communities in achieving balance of the body, mind, spirit, and emotion [20,21,22]. In responding to Call to Action #33, future research and prevention efforts must be re-centered around Indigenous knowledges and worldviews in order to redress experiences of intergenerational trauma and support self-determination—giving individuals a sense of belonging, hope, and a greater understanding of their meaning and purpose of life, all of which are integral to Indigenous wellness [19,22,23].

*Nimi Icinohabi* (Our Way of Life) and LIVE (Living Indigenous Values Everyday) are two projects initiated by the Alexis Nakota Sioux Nation (ANSN) with the guidance of the *Ish?awimin* (Elders) and by kinship relations, as rooted in language, culture, and spirit. ANSN is the most northwesterly group of Siouian speaking peoples in North America, located on the shores of *Wakamne* (Lake that is Sacred/Lac St. Anne) in Alberta.

The *Nimi Icinohabi* program (2007–2011) began as a school-based culturally adapted prevention program aimed at addressing the rise of children born with FASD and was further adapted and delivered to young women and men in the community. During the development of the *Nimi Icinohabi* program, the Nakota *Ish?awimin* emphasized the importance of honouring the connection of *wahogicobi* (kinship relations) to *waka* (Sacred), *doshgamin* (children), and *xamcashdabi* (People of the Land) [24]. The program was facilitated by community members fluent in the Nakota language who understood the cultural and spiritual importance of honouring *wahogicobi* as a way of life.

The program coordinator described the importance of centering prevention around the knowledge of their Elders: “We found that while we might have carried out the research without the involvement of the *Ish?awimin*, the project would not have resulted in the depth and meaning experienced by all involved. At the conclusion of the Nimi Icinohabi program, the *Ish?awamin* emphasized the need to have a similar program for the parents whom they described as, ‘the generation who missed out on these teachings.’”

LIVE was subsequently developed by ANSN community members with the Alberta Northwest Central FASD Network using the *Ish?awimin*’s teachings and lessons learned from *Nimi Icinohabi* to create a collaborative approach to *ugicigebicen* (help each other)*, nagicishbabicen* (break the cycle) and lead the path to nurtured families [25]. Led by a highly regarded knowledge keeper and an active working group that included community-based and outside agency partners, the program uses a decolonizing approach to engage in community development and provide community education on FASD with the provision of culturally specific supports to individuals with FASD and their families.

Emphasis was placed on Nakota identity through history, culture, language and spirit, as all connected to wellbeing. The value of recognizing each person’s gifts regardless of their challenges, through kinship and other cultural teachings, re-centres relationships, and facilitates more effective service delivery. It dually provides opportunities to ‘re-story’; through the acknowledgment of the historical impacts on the community and the provision of a foundation to support community members with FASD, their families, and the community through the values taught by the *Ish?awimin*.

#### 3.1.2. Tenet #2: Using a Social and Structural Determinants of Health Lens

Prevention and research programs informed by the social and structural determinants of health go beyond addressing individual behaviour to positively support women’s wellness and healing, and that of their families and communities [22,26]. Engaging with the social and structural determinants of health re-centres conversations about alcohol and other substance use to focus on wellness. A determinants-based approach is also concerned with how the intergenerational effects of colonization and the residential school system continue to be experienced and cause barriers to accessing health and social services. Acknowledging the ongoing effects of colonization removes stigma and blame from the individual, and can better assist women, families, and communities to access wellness and healing oriented supports [19,26,27].

FASD prevention programs that are community-led and community-driven are often built to respond to the geography, infrastructure, language, culture, and human resource capacity that support engagement in services. They can also respond to structural barriers, such as systemic and institutional racism resulting in disrespectful treatment towards Indigenous women, girls, and their families [28].

The Circle of Life Mentorship Program (COL) is operated out of the Kermode Friendship Society in Terrace, British Columbia on the Tsimshian Territory. The program serves urban Indigenous and non-Indigenous families of childbearing years from various Nations surrounding the Kermode Friendship Society.

The program is based on the Parent Child Assistance Program (PCAP), a home visitation/case management program which began in 1991 at the University of Washington, USA [29]. The program is designed to empower women to make healthy life changes and decrease the number of alcohol exposed pregnancies through mentorship over a three-year period.

COL uses trauma-informed and harm reduction approaches to create a safe and supportive environment for each individual, their family, and their support network to increase their self-determination and embrace their knowledge and culture. During the three-year program, COL aims to create positive outcomes for the individual and family unit by working with both parents to create attainable goals and collaborate with key people in their lives, such as their extended family unit and support workers, to help achieve their goals.

Mentors also act as health navigators, providing referrals to health and community services and providing opportunities for skill-building related to budgeting, parenting, and family planning, or anything else that women, their partners, and support network identify as important to reaching their goals. Working with women to overcome pervasive barriers to health and service access, such as housing or food security as key initial and foundational steps before focusing reducing substance use, has been important to FASD prevention. Interventions that address social and environmental factors that affect a woman’s ability to decrease substance use, as well as the reasons that may influence their substance use, are often more effective [30,31,32].

The current and former program managers describe the uniqueness of the program and the approaches that they use:

“We know that traditionally, in our Indigenous culture, each member in a family unit has a role to play and we strive to convey their strengths as interwoven with Westernized practices and protocols. Our goal is to help alleviate any barriers to success that the family unit faces and to strengthen their identity and community wellness. Therefore, we acknowledge the importance and strength of the medicine wheel and the holistic focus of a family. By integrating partners into the program, we are able to empower the father to quit drinking, which is just as important as the mother quitting. We look also to support our clients and help them problem solve. For women who are struggling to reduce their use, we might ask her and her partner to focus on having a drink of water between each alcoholic drink, focus on eating healthy food, and connecting with a family doctor throughout the pregnancy. These are all ways to support our clients and help families feel success rather than judgement.”

The community leans on COL and the Kermode Friendship Society to help address gaps in services that families face. This has increased service provider capacity as well through giving providers a better understanding of how complex FASD is.

#### 3.1.3. Tenet #3: Highlighting Relationships

FASD prevention programs must acknowledge and support the role that extended family has in child development and socialization. When Elders, community members, and extended family members share their knowledge about pregnancy, childbirth, and mothering, essential components of Indigenous language and culture are preserved [1,2]. Building prevention programs that preserve and highlight familial and other relationships can further integrate culture into practice and can further the development of respectful relationships between community members, researchers, and service providers [2,12,19].

Indigenous concepts of wellness, healing, and health are holistic and frequently emphasize the importance of each person’s relationship to their family and community, the environment, and the Creator. The two Innu First Nations in Labrador developed a Guide to the Innu Care Approach that articulates:

“It is also clear that the effective ways to face and overcome these challenges come from the strength and resilience of Innu culture and relationships…The true experts in Innu culture, Innu-*aitun*, are the Innu.

…families and communities that have been impacted by trauma face greater challenges in providing the necessary support for growing children. In that context, bringing the best of our qualities together is even more important. For Innu, this includes our respect for one another, our capacity to work together in the best interests of our children, our ability to trust and depend on one another, our love and value for family, and our timeless relationship to *Nutshimit* [the land], the source of all our health and wellbeing” [33] (p. 2).

The Innu Care Approach is, at its core, about healing a nation and investing in children, to ensure healthy and thriving Innu people, communities, and culture for many generations to come. The Innu Care Approach uses the image of a teepee-like structure with poles, called *tshuap* poles, to name the essential supports and relationships needed for children to thrive which include parents, extended family, community, Innu-governed services, Innu culture and language, and Elders. Furthermore, if people from outside the Innu communities are involved in FASD prevention and respect the communities’ rights to self-determination, they must be invited. And to be invited, relationships built on trust and respect for the Innu, the land, and the culture must be nurtured.

#### 3.1.4. Tenet #4: Community-Based and Community-Driven

The residential school system and subsequent assimilatory policies and practices contributed to the breakdown of family and community relationships, and loss of traditional parenting knowledge [1,12]. While the experiences of intergenerational trauma are shared [10], experiences differ across nations, peoples, and communities. Indigenous peoples and nations are distinct and diverse: First Nations, Métis, and Inuit communities have unique histories, languages, worldviews, cultural practices, and spiritual beliefs [19,34]. Therefore, FASD research and prevention programs need to build upon the specific community’s interests, needs, wisdom, language, and knowledge [35]. By providing community-based care, providers can ensure that the community’s worldviews and concepts of health, healing, and wellness are central to service provision and support. Community-based and driven services can recognize historical relationships, integrating the community’s strength and culture to improve capacity to respond to the needs and realities of community members [2].

Anti-colonial and decolonizing approaches to governance, service provision and research inherently requires valuing Indigenous people’s rights to self-determination. A participant from Skidegate on Haida Gwaii Island, British Columbia spoke of developing FASD prevention programming and noted the importance of developing community-based, community-driven programs:

“The first question I always ask myself is ’what does this community need?’, from there I find the supports and resources. My community is on the edge of the world; the standing joke is taking the ferry over to Canada. It does sum up what it’s like to live in a rural and remote community. We don’t have access to many professionals—they usually fly in and out every few months—or the resources that many others take for granted. Many of us doing the job wear several hats.”

In their program, it is integral that all of the work is not only community-driven, but also relationship-based. As the participant noted, “without relationships, nothing can happen in a healthy and positive way.” To develop FASD prevention, pregnancy, or parenting programs, the Skidegate Health Centre incorporates the mother, the family and the community. Developing FASD prevention programs requires supporting clients and community members where they are at, without judgement, and finding teachable moments that meet their needs.

#### 3.1.5. Tenet #5: Provision of Wraparound Support and Holistic Services

Wraparound support involves coordination across community supports and services to meet the complex emotional, mental, physical, and spiritual needs of women and families [36]. The use of wraparound supports, including the use of pre-existing supports from the community and extended family can tailor care to the mother’s and child’s needs and build on the women’s and community’s strengths [2,3]. Through building on individual and collective strengths, wraparound support can also play a role in increasing individuals’ self-determination and prevent further trauma [11]. Focusing on the provision of wraparound support allows community members and prevention staff to move beyond the narrow focus of individual behaviours and address intersecting determinants of health that lead to meaningful and sustainable outcomes for both family and community [19,36].

Culturally-informed programs that are holistic and provide wraparound support can also be provided in urban contexts through outreach and ‘one-stop’ drop-in services. *Manito Ikwe Kagiikwe* (The Mothering Project) located in Winnipeg, Manitoba is a FASD prevention program that adopts a harm reduction and trauma-informed approach, with a focus of culture integrated in all of its programming. The program supports women who are pregnant, parenting, or who have children living in foster care or with extended family. *Manito Ikwe Kagiikwe* is based out of Mount Carmel Clinic, a health centre, and has a licensed daycare program for infants which offers subsidized spots for infants and preschool aged children. The program also provides substance use and nutritional services, culturally-informed counselling, and assistance to families in navigating systems of care. Women are invited to participate in programs and events related to their culture, such as smudging, a drumming circle, baby naming, and group programming. Having access to co-located health and social services has helped women in meeting their diverse needs and has supported several mothers to keep their children in their care. [18].

#### 3.1.6. Tenet #6: Adopting a Life Course Approach

For FASD prevention, adopting a life course approach recognizes the historical and cultural role of pregnancy and child birth as a sacred part of the life cycle. [2,37,38]. Re-centering service delivery around Indigenous worldviews connects individuals to their culture and purpose, as articulated in the Wellness Framework [2,20]. Moreover, it promotes positive parenting and reinforces familial and community roles to raising children in ways that disrupt intergenerational trauma [11,19,26].

The Stó:lō Family Empowerment Team (FET) has created a culturally safe, relationship-first program for urban and rural Indigenous females. The program mandate is to decrease the incidence of FASD in the eleven Stó:lō communities. FET is based upon the Indigenized PCAP and it includes additional and complementary programs including Growing Great Kids Parenting Program, Stó:lō focused traditional parenting, Indigenous doula services, and a care committee for child welfare advocacy.

The current and former program managers shared:

“The Stó:lō Elders gifted the program a traditional name in *Halq’eméylem* (language of S’olh Temexw, traditional territory of the Stó:lō people) of *Xyólhmettsel Syémyem*, meaning I am taking care of my pregnancy. The word *Xyólhmettsel* indicates a caring and loving relationship and was chosen specifically to reflect the relationship between a caretaker and receiver—the participant and child. This relationship between the client and advocate share is sacred and must be based on what the client needs in whichever way the client can comfortably engage in.”

The measured outcomes of FET demonstrate the importance of building programs using Indigenous and relationship-first approaches and modern health tools that support individuals across their life course in achieving wellness outcomes.

#### 3.1.7. Tenet #7: Models Supporting Resiliency for Women, Families, and Communities

Models supporting resiliency, including the use of strengths-based approaches that situate Indigenous culture as foundational to program planning and implementation, are integral to wellness and self-determination for women and their families [7,21,27,34,37]. Strengths-based models avoid defining individuals through labels such as “they’re FASD” or stigmatizing claims that FASD is “100 percent preventable”. Through the use of strengths-based models, FASD prevention researchers and frontline workers avoid punitive and shaming approaches, and deficit-oriented models of substance use problems. Resiliency and strengths-based approaches empower women in their role as mothers and foster maternal-child attachment. They are guided by the concepts of wellness and health promotion; encouraging women to stop or reduce alcohol use while preparing for pregnancy, during pregnancy and when breastfeeding. They encourage families to see their strengths and resource capacity, and engender hope, belonging, meaning and purpose [19].

The Lakeland Centre for FASD (LCFASD) has been able to offer resiliency-oriented prevention, diagnosis, and intervention support programs with and within First Nations and Métis communities in northeast Alberta. Each program partners with a variety of groups within the respective communities to offer group learning sessions, outreach support, and diagnostic clinics. The LCFASD recognizes that each community is unique and works to build relationships with services and individuals that are wanting to address women’s resiliency, skill building, cultural teachings, healthy pregnancy and parenting. The LCFASD’s aim is to assist individuals and families to be good advocates for the services that they need, and to be able to identify and draw upon their strengths.

The LCFASD offers an array of programs, but one way that they have facilitated this work is through jointly offering a women’s resiliency group with a nearby Métis Settlement. The group covers topics of safety, traditional food preparation, cultural child care, pregnancy health and addictions support. The group was so successful that the community began running the group continuously with minimal support from the LCFASD. The program has now been adopted by several other communities.

#### 3.1.8. Tenet #8: Ensuring Long-Term Sustainable Funding and Research

Currently, Indigenous worldviews and knowledge systems are not adequately recognized in research and service delivery [2,16]. Consequently, community leaders, elders, knowledge keepers, and FASD prevention experts have identified the need to address structural and systemic change through decolonizing research methods. Research priorities must be determined by Indigenous communities and groups; so that knowledge held within communities, languages, and cultures are incorporated in research practice [2,35,37,39]. In order to effect change, funders must recognize the scope of FASD prevention and the work involved in delivering comprehensive FASD prevention programs to ensure that Indigenous-led prevention programs and research are well-funded and sustained. This ensures forward movement in the decolonization of research and the quality of life of Indigenous peoples [19].

To address the need for adequate and sustained funding and to ensure that health and social programs do not cause harm to the Indigenous peoples and communities that the programs serve, an ethical policy framework is needed [40]. As Tait (2008, 2012) and other researchers have emphasized, the implementation of ethical frameworks for funding, program development, research, and evaluation must take place through Indigenous self-determined processes and through partnerships between Indigenous and non-Indigenous governments, funders and researchers [40,41]. Community-based evaluations also need to reflect and incorporate the perspectives of Indigenous communities and peoples being served [42,43].

Such approaches have been echoed by researchers who collaboratively evaluated the implementation of the PCAP in First Nations communities in Alberta [44,45]. One researcher involved in the evaluation shared:

“The prevention staff in these diverse communities shared how they built upon PCAP tenets to respectfully deliver prevention services. The mentors worked extensively outside of their job descriptions to meet the needs of their communities through strong relational practice with the rewarding outcomes of improved individual and familial wellbeing, and community-wide impacts of increased knowledge about and decreased stigma around FASD. This work was time intensive and mentors relayed the importance of shifting towards longer-term funding models that demonstrate forward movement toward reconciliation and change.”

Another evaluation, conducted by Tait (2008), further emphasized the need for long-term sustainable funding:

“Based upon scientific evidence, three-year P-CAP programs have successfully reduced rates of FASD, improved maternal health… and reduced health and social services costs in the form of services for children with FASD and women with substance dependencies. However, my evaluation found that without appropriate levels of support—financial, time, and human resources—these benefits were reduced to a minimum, despite the committed efforts of local mentors and their communities.” [46] (p. 55).

Implementing ethical frameworks for funding, program development, research, and evaluation is integral to appropriately recognizing the time and effort required to ensure the establishment and delivery of prevention programming. This will require a responsive call for a change in program, research, and evaluation funding practices, and collaborative and participatory research and evaluation which honour the importance of relational practices with Indigenous peoples and communities.

## 4. Discussion

To enact Call to Action #33, effort is required to move from the Western medical paradigm with its focus on treating alcohol and other substance use concerns, to an integrated approach that is wellness and life course oriented and recognizes how the social determinants impact health and substance use [38,47]. The eight tenets of the Consensus Statement and the exemplary FASD prevention programs emphasize that health education about the dangers of alcohol use during pregnancy should not be the primary focus of FASD prevention. Rather, FASD prevention in Indigenous communities must respond to the historical, social, political, economic, and cultural dimensions of alcohol use and misuse during pregnancy and across the lifespan. Similar findings have been offered by Gonzales et al. (2018) on the importance of addressing historical, contemporary, and intergenerational healing and resilience in creating effective, multi-level FASD prevention programs in Portland, Oregon, USA [11].

The Consensus Statement emphasizes the importance of supporting a strengths-based approach to FASD prevention by focusing on culture and relationships as the foundation to building resilience and self-determination at the individual, family and community levels. In all of the programs, cultural connection plays a central component to healing and self-determination. This can be equally important for Indigenous peoples living in urban centres who, through holistic FASD prevention efforts are able to (re)engage in cultural learning in order to support their wellness.

The Consensus Statement can guide other initiatives within the maternal child health field that may not consider FASD prevention as their primary mandate. When broader maternal child health initiatives in Indigenous communities facilitate change and healing in a way that has the potential to support Indigenous women in giving birth to healthy children, address the intergenerational transmission of trauma that disrupted positive parenting and family relationships, and actively contribute to the ongoing process of reconciliation; they become a form of FASD prevention.

The eight programs highlighted in this article were developed interdependently of the Consensus Statement. However, each program highlights one or more of the tenets of the Consensus Statement in their work, which is demonstrative of the importance of programming that addresses women’s and their families’ health, social, and cultural needs as guided by elders, knowledge keepers, and extended families. The diversity of these FASD prevention programs is representative of the diversity of Indigenous peoples and the vastness of how community-driven FASD prevention approaches can be developed and implemented in a contextual, collaborative, and reconciliatory manner to respond to Call to Action #33.

Using the Consensus Statement as a guiding document and *Etuaptmumk* as a guiding approach emphasizes the importance of interdisciplinary and reconciliatory Indigenous FASD prevention research agendas being co-developed, and that priority is given to supporting and creating ongoing opportunities for collaboration across communities. As the Consensus Statement and the eight exemplary programs demonstrate, expertise is propelled through relationship-building and knowledge-sharing opportunities.

Overall, implementing Call to Action #33 requires an ongoing and collaborative commitment to reconciliation; recognizing Indigenous communities’ rights to self-determination, supporting healing relative to recognition of past harms, and acknowledging the ongoing impact and legacy of colonialism. Implementing Call to Action #33 also involves facilitating collaborative and dialogue-driven action, such as the meetings underlying this article, with their hope that political will and commitment to bring about paradigm shifts and systemic change will follow [9].

## 5. Conclusions

Community-based, culture-led FASD prevention initiatives have emerged in Indigenous communities across Canada. Researchers, community-based advocates, program planners, leaders, and knowledge keepers have gathered to address and document the complexities of FASD prevention—work that involves addressing the topics of mothering, alcohol use, and social and structural determinants of health affecting Indigenous communities in Canada.

While FASD prevention work in Indigenous communities has not always been evaluated and otherwise evidenced [48], we are now seeing the development of diverse, culture-led initiatives and an interest in collectively documenting the strengths and challenges of this work. These initiatives need to be noticed, even if they are not being specifically named FASD prevention initiatives, as many of the initiatives address the broad social and structural determinants of health that are associated with alcohol use in pregnancy. Such approaches are consistent with the Consensus Statement and holistic and strengths-based Indigenous worldviews, which have been critical to advancing FASD prevention work in meaningful ways.

We advocate for the eight tenets in the Consensus Statement to be further drawn upon as promising practices for FASD prevention in Indigenous communities. Moving forward, we urge FASD prevention researchers, elders, knowledge keepers, program planners, and community leaders to engage with the eight tenets and share successes and challenges associated with community-specific approaches to FASD prevention.
